# Designing of multi-objective optimal virtual power plant model for reliability enhancement in radial network: a case study of Indian power sector

**DOI:** 10.1038/s41598-022-16389-8

**Published:** 2022-08-04

**Authors:** Harpreet Sharma, Akmaral Imanbayeva

**Affiliations:** 1 Northeastern University, Toronto, Canada; 2 IETP AI—Farabi Kazakh National University, Almaty, Kazakhstan

**Keywords:** Engineering, Electrical and electronic engineering

## Abstract

One of the major driving factors in the shifting of the present grid paradigm to an active grid network is the reliability and resiliency of the utility network. With hefty investment in the distribution network protection and maintenance, the reliability of the feeders is considerably enhanced; however, large numbers of outages are still occurring every year which caused major production loss to the manufacturing sector. In this paper, the role of the solar grid-based Virtual Power Plant (VPP) is evaluated in the state power utility for the reliability enhancement and cost minimization using a multi-objective model based on MILP optimization. A 90 bus industrial feeder having automatic reclosers, DER, and DSM is selected on which the MCS method is utilized for computing reliability indices using the utility reliability parameters. The value of reliability indices such as EENS is declined by 68% by utilizing the VPP scenario. These values of this reliability index are fed into the multi-objective model for cost minimization. After running the optimization, the results reveal that the operational and the annual energy cost are reduced by 61% and 55% respectively which advocates the VPP implementation in the utility network. Both modes of the Virtual Power Plant such as grid-connected and autonomous mode have been discussed in detail. Lastly, the results of the developed model with MILP are compared with the proprietary derivative algorithm, and it is found that the proposed MILP is more cost-effective. The overall results advocate the VPP implementation in the utility grid as the economical advantage is provided to both utility and the consumers in terms of reduction in EENS and energy charges respectively.

## Introduction

The smart grid revolutionizes the present grid infrastructure with its large-scale integration of small rating grid-connected DERs installed at the consumer premises. The power generation from these DERs is often clean and low cost, especially in the case of renewable-based DER such as roof-top PVs which gives hopes of a sustainable energy future as well. However, there are still some issues associated with DER power generation which limit its application in reliability improvement^1–4^.

The theory of VPP takes full advantage of DER by integrating their generating profiles into a single operating profile which is easy to control and dispatch^5–7^. During the unscheduled outage due to any fault in the radial network, the entire grid-connected consumers are at the risk of interruption which results in production loss and adversely affects the national economy. In the present grid infrastructure, the automatic reclosers are mostly used in the radial feeder for isolation of faulty sections during an outage and assist in restoring the supply of the remaining consumers under a healthy portion of the feeder. The application of automatic recloser slightly enhances the overall system reliability; however, to get substantial improvements, the concept of VPP needs to be implemented in the distribution network. The power supply can be restored through the VPP during an outage by creating intentional islanding through the reclosers and dispatch of an aggregate generation of rooftop PVs and with the shifting or interrupting a load of low priority as DSM^8^. This strategy makes it possible to meet emergency load in case of grid unavailability and even reduces the load stress during feeder reconfiguration. The interconnected DERs and DSM techniques can only be put into operation through automatic recloser as during fault the unhealthy section must be isolated from the healthy portion. The remaining faulty portion can be energized through the VPP and the main grid which further depends upon the fault location^9^. The generation profiles of DERs are integrated into one operating configuration and load is dispatched securely by optimal control strategy. To calculate the reliability, the analytical method is undoubtedly simple with less computation time but it lacks accuracy due to its fixed average value which limits its use in practical applications. The MCS approach provides numerous possible values through its probability distribution and gives realistic results in comparison to the simple analytical method as the utility distribution system is stochastic^10^. The MCS predicts the behavior of the system accurately and is classified into two categories: sequential and non-sequential. The sequential MCS is used in this study as it simulates the system in the chronological order of time and models the system components more realistic with the time-varying loads. The various reliability indices are calculated after simulating the artificial failure history of the various components and overall system reliability is determined. On the other hand, for the optimal scheduling for minimization of cost and reliability enhancement, the MILP is the effective optimization technique for accurate modeling of the sophisticated system of the VPP in comparison to the other available techniques.

### Literature review

The ETAP software is used in a standard RBTS network^11^ for enhancement of the system reliability with DG injection at different points. In similar research in^12^, the utility case study is taken into account to determine the effect of multiple DG on grid reliability and power loss using Matlab and DIgSILENT, without calculating the financial implications of DG on the distribution network. The reliability enhancement of buildings with the solar installation using energy storage like batteries and EV is proposed in^13^ without interlinking with the utility grid. In the study^14^ for reliability improvement and loss reduction, an optimal solution of DER placement based on the performance index such as total energy consumed and energy not supplied is introduced. This research does not consider the multiple DG influences on reliability. The multi-objective function is introduced in^15^ for the reduction in power loss and reliability enhancement with DGs by the application of dynamic programming. However, while enhancing reliability, probabilistic ways such as MCS are also not utilized for system failure calculation. The transformer’s reliability improvement is analyzed in^16^ with high penetration of DERs such as diesel generators, PV, and wind. But overall system reliability indices are not evaluated and economic analysis has not been done. In^3^ the genetic algorithm embedded Monte Carlo Simulation is applied on 15 bus and 33 bus radial feeders to meet the growing load demand and enhance the reliability of the network. In this research, there is no attention given to system overall reliability enhancement and the effect of multiple located DGs as VPP is not evaluated. The study^17^ calculates the various reliability indices of the IEEE RBTS integrating DERs using the Markov model which is not efficient for a large network and there is no provision of multiple DGs. In a similar study using the VPP scenario in^18^, the ETAP software is used to evaluate the reliability of the IEEE RBTS integrating DERs at one or multiple locations but the financial aspects of these improvements are not taken into account. In^19^ the study relates to the optimal allocation of DR with the utilization of other smart grid technologies for the reliability enhancement is proposed without considering DER. The Markov approach is followed in^20^ for standalone hybrid energy systems in computing the reliability indices without emphasizing the financial implication and there is no DSM technique has been used. The adverse effects of high penetration of DER in the grid are addressed by the DR in^21^ without the VPP scenario. In^22^ the multi-agent ontology-driven energy management is proposed for combined dispatch of DER, DR, and battery for cost minimization in the microgrid framework; however, there is no consideration is given to the reliability implication and utility influence in the VPP framework. The study^23^ proposes the PSO for optimal scheduling of reconfigurable microgrids having various DERs without implementing DR and calculating reliability influence. The machine learning also find application in the VPP for optimal scheduling and understanding the load and generation pattern in^24,25^. The flexible renewable VPP utilized as a two layer model on IEEE 69 bus distribution network is proposed in^26^, however the realistic study is still lacking. In^27^ a novel application of Data analytics as Deep learning is proposed in the multi energy system VPP with various energy resources for the optimal scheduling. The optimal dispatch model based stochastic game approach is utilized on the real case studies in^28,29^ without influence of the DR. Another study^30^ used the battery energy system for managing high penetration of the renewable share in the VPP without DR.

### Novelty and contribution

The present studies assessed the reliability improvement with DER penetration and limited the location of DER in the standard test feeders, which does not present the real-time characteristics of the system. Moreover, less emphasis is given to the financial implications of reliability enhancement with DERs. As per our knowledge, any techno-economic study based on the VPP concept of multiple DER and DSM for reliability enhancement is not reported so far. The main motive of this paper is to fill the research gap of the previous studies and to develop the multi-objective VPP model based on MILP optimization. The model permits the VPP optimal scheduling for cost minimization in grid-connected mode and reliability improvement in autonomous mode during an outage. The reliability of the system is enhanced with the inclusion of a network of small-scale rooftop PVs and the application of DSM techniques such as load shifting and shedding. The probabilistic approach such as MCS is utilized to compute the reliability indices with DER penetration. These reliability indices fed to a multi-objective optimal model for energy scheduling and techno-economical analysis has been done.

### Paper organization

The rest of the paper is described as follows: In “Reliability analysis”, the methods of reliability analysis are explained including the MCS method for calculating reliability indices. “VPP mathematical modeling” gives a brief description of the VPP mathematical modelling and its scheduling based on MILP. “Utility case study” elaborates on the case study of the utility parameters including its resource and technical specifications In “Results and discussions” a detailed description of the results of the MCS and multi-objective model are given. “Conclusion” concludes the major results of the research and the policy recommendations are given.

## Reliability analysis

To assess the utility reliability, the reliability indices are calculated from the system parameters such as the number of customers, interrupted customers and duration of the interruption^12,17,31^.

### Reliability indices

#### System average interruption frequency index (SAIFI)

It reveals the frequency of an average customer interruption for a specific course of time. The SAIFI is given as

$$\mathrm{SAIFI }= \frac{\mathrm{Total} \, \mathrm{ Number} \, \mathrm{ of} \, \mathrm{ Customers} \, \mathrm{ Interrupted}}{\mathrm{Total } \, \mathrm{Number} \, \mathrm{ of} \, \mathrm{ Customer} \, \mathrm{ Served}}$$

The isolation of a faulty section of the feeder through recloser, can assist in restoring the supply of a healthy portion and declines the number of customers interrupted.

#### System average interruption duration index (SAIDI)

It shows the duration of the interruption that an average customer faced in the specified course of time. The SAIDI is given as

$$\mathrm{SAIDI }= \frac{\mathrm{Customer } \, \mathrm{Interrupted} \, \mathrm{ Duration}}{\mathrm{Total} \, \mathrm{ Number} \, \mathrm{ of } \, \mathrm{Customer } \, \mathrm{Served}}$$

The duration of interruption could be reduced with the reclosers for fault isolation and DER for supplying the healthy portion.

#### Customer average interruption duration index (CAIDI)

This reveals the average time needed to restore the supply. The CAIDI is given as

$$\mathrm{CAIDI }= \frac{\mathrm{Customer } \, \mathrm{Interrupted } \, \mathrm{Duration}}{\mathrm{Total } \, \mathrm{Number } \, \mathrm{of } \, \mathrm{Customer} \, \mathrm{ Interrupted}}$$

With the fixed number of interrupted consumers, the CAIDI can be reduced with the restoring time by supplying the isolated section of the feeder by the DER.

#### Average service availability index (ASAI)

It indicates the percentage of duration in which an average customer received the electrical supply in the specified course of time. The ASAI is given as

$$\mathrm{ASAI }= \frac{\mathrm{Customer } \, \mathrm{Hours } \, \mathrm{Service} \, \mathrm{ Availability}}{\mathrm{Customer} \, \mathrm{ Hours} \, \mathrm{ Service } \, \mathrm{Demands}}$$

The supply of demand through the DER during the fault period can enhance the service availability of the customers and ultimately the ASAI index.

#### Expected energy not supplied (EENS)

It depicts the average electrical demand in the year which is not met by the system. It is given as

$$\mathrm{EENS }=\mathrm{ Average } \, \mathrm{Outage} \, \mathrm{ Time}*\mathrm{Total } \, \mathrm{Electrical } \, \mathrm{Demand}(\mathrm{kWh }/\mathrm{ Yr})$$

The demand is dispatched from the aggregated DER and the main grid after isolating the faulty section can be reduced EENS.

The methods of calculating reliability indices are classified into two major categories: analytical and MCS.

### By analytical method

This is one of the simplest and fastest methods available for computing reliability indices of distribution networks. In this method, the impact of each component failure on the load points is calculated to determine the frequency and duration of the interruption. The values computed from this method are mean values that don’t have much practical significance as the behavior of the utility network is stochastic and the results obtained from the analytical method are impractical.

### By MCS method

In MCS, the sequential method is mostly utilized by researchers due to its numerous advantages. In the sequential method, the probability distribution is calculated which indicates the close range of the reliability indices. The various characteristics of the system are modeled in chronological order in the form of an up or down cycle. Sequential is the only method, which can model time-varying loads.

The basic parts of the MCS method are further elaborated as follows:

#### Artificial operating history

The generation of components failure history is the basic requirement of MCS. The artificial operating history can be generated by defining the reliability parameters such as failure rate and MTTR of the specific component. The artificial failure history generation is the dual-state model which means the component stays either in UP state called Time To Failure (TTF) or in downstate which is known as Time To Repair (TTR)^32,33^. The switching time between these states is assumed to be zero. In the Matlab environment, the random numbers between 0 and 1 are generated which represents a component failure/working in the exponential distribution^16,34,35^. The calculation of TTF and TTR is given as follows:

$${\mathrm{TTF}}_{\mathrm{i}}=-\frac{\mathrm{ln}\left({\mathrm{U}}_{\mathrm{i}}\right)}{{\uplambda }_{\mathrm{i}}}\times 8760 \,h$$

$${\mathrm{TTR}}_{\mathrm{i}}=-\mathrm{ln}({\mathrm{U}}_{\mathrm{i}})\times {\mathrm{MTTR}}_{\mathrm{i}}\,\mathrm{ h}$$

The Table A1 shows as component wise failure data of the industrial feeder as a supplementary data.

#### Customers interruption

The system response to the faults that are generated during the artificial failure history indicates the number of customers is interrupted due to the specific component failure in that region. The every hour system is assessed for possible faults and their consequence interruption considering the application of reclosers and DER.

#### System islanding

In the event of a grid outage, the customers connected to the particular portion of the system faced interruption. However, the supply can be restored by intentional islanding with the implementation of the VPP concept^36,37^. The faulty area of the feeder is isolated with the help of automatic reclosers and the healthy portion is supplied by DER and the unmet load is either shifted or curtailed.

## VPP mathematical modeling

The DER-CAM model is designed by Lawrence Berkeley National Laboratory, USA applied to find the optimal investment solution and DER dispatch^7,38^. DER-CAM finds its key application in VPP and microgrid operations specifically in the planning phase. The base of the DER CAM optimization process is MILP.

**Load parameters**

- $${\mathrm{U}}_{\mathrm{Loadt}}^{\mathrm{Imp}}$$Energy imported from the grid (kWh)
- $${\mathrm{C}}_{\mathrm{EENS}}$$Cost of not supplying demand ($/kWh)
- $$\mathrm{EENS}$$ Expected Energy Not Supplied (kWh)

**Tariff details**

- $$\mathrm{Ttod}$$ Energy import charges for time of day [$/kWh]
- $$\mathrm{TEx}$$ Energy export charges for time of day [$/kWh]
- $${\mathrm{An}}_{\mathrm{i}}$$ Annuity factor for investments in technologies i
- $${\mathrm{TF}}_{\mathrm{m}}$$Tariff for fixed charges for using utility infrastructure in month m [$]
- $${\mathrm{TP}}_{\mathrm{s},\mathrm{p}}$$ Maximum demand charges under the PSPCL tariff for season s and period p [$/kW]
- p Tariff period (on-peak, mid-peak, off-peak)
- s Season (winter, summer including paddy season)

**Technical parameters of solar PV**

- *Max*S Maximum rating of solar PV [kW]
- *OM*C Fixed annual O&M costs of solar PV [$/kW]
- *MaxH* Maximum annual operation hours for technology [hour]
- $$\mathrm{SPE}$$ Theoretical peak solar conversion efficiency [%]
- $${\mathrm{SRE}}_{\mathrm{m}.\mathrm{h}}$$ Solar radiation conversion efficiency of generation technology c, in month m, and hour h [%]
- $${\mathrm{SI}}_{\mathrm{m},\mathrm{d},\mathrm{h}}$$ Solar insolation during the specified period of a month, day and hour [kW / $${\mathrm{m}}^{2}$$]
- $$\mathrm{SA}$$ available area for solar technologies [m^2^]
- $$\mathrm{FCC}$$ fixed capital cost of solar PV[$]
- $$\mathrm{VCC}$$ variable capital cost of solar PV in [$/kW]

**Decision variables**

- NS Number of units of solar PV installed
- ${\mathrm{PV}}_{\mathrm{t}}$Power generated by solar PV [kW]
- *Cap* Rated output of solar PV [kW]
- ${\mathrm{U}}_{\mathrm{Loadt}}^{\mathrm{Imp}}$ Electricity purchased from utility [kW]
- *Pur* Consumer purchase binary decision of solar PV [0 or 1]
- ${\mathrm{U}}_{\mathrm{Loadt}}^{\mathrm{Exp}}$ Electricity exported to the utility [kW]

**Objective function**

1 $${\text{MinC}} = \Sigma _{{\text{m}}} {\text{TF}}_{{\text{m}}} + \Sigma {\text{U}}_{{{\text{Loadt}}}}^{{{\text{Imp}}}} \cdot {\text{Ttod}} + \Sigma _{{\text{m}}} \Sigma _{{{\text{m}} \in {\text{s}}}} \Sigma _{{\text{p}}} {\text{TP}}_{{{\text{s}},{\text{p}}}} \cdot {\text{max}}(\Sigma _{{{\text{u}} \in {\text{eo}}}} \Sigma {\text{U}}_{{{\text{Loadtm}},\left( {{\text{d}},{\text{h}}} \right) \in {\text{p}}}}^{{{\text{Imp}}}} ) + \Sigma ({\text{FCC}} \cdot {\text{Pur}} + {\text{VCC}} \cdot {\text{Cap}}) \cdot {\text{An}} + {\text{Cap}}.{\text{OMC}}?\Sigma {\text{U}}_{{{\text{Loadt}}}}^{{{\text{Exp}}}} .{\text{TEx}} + \Sigma {\text{EENS}}.{\text{C}}_{{{\text{EENS}}}}$$

**Network constraints**

- $${\mathrm{Load}}_{\mathrm{t}}=\Sigma {\mathrm{PV}}_{\mathrm{t}}+{\mathrm{U}}_{\mathrm{Loadt}}^{\mathrm{Imp}}[\mathrm{kW}]$$ (2)
- $${\Sigma (\mathrm{\Sigma PV}}_{\mathrm{t}}+{\mathrm{U}}_{\mathrm{Loadt}}^{\mathrm{Exp}})\le \mathrm{NS MaxS}\cdot \mathrm{ MaxH }[\mathrm{kW}]$$ (3)
- *Cap* ≤ *Pur*∙ *M* [kW] (4)
- $${\Sigma }_{\mathrm{u}}{\mathrm{PV}}_{\mathrm{t}}$$+ $${\mathrm{U}}_{\mathrm{Loadt}}^{\mathrm{Exp}}$$  ≤ $$\mathrm{Cap}$$∙$$\frac{\mathrm{SRE}}{\mathrm{SPE}}$$
- (6)
- $$\mathrm{EENS}={\mathrm{Load}}_{\mathrm{t}}-{\mathrm{FlexL}}_{\mathrm{t}}-{\mathrm{IntrrL}}_{\mathrm{t}}-{\mathrm{PV}}_{\mathrm{t}}$$ (7)

Equation (1) explains the objective function for minimization of annualized cost and includes all the economic components like DER capital cost, operation cost, utility charges, etc. In objective function $({\Sigma }_{\mathrm{m}}{\mathrm{TF}}_{\mathrm{m}}$) represents fixed charges for the month m, $(\Sigma {\mathrm{U}}_{\mathrm{Loadt}}^{\mathrm{Imp}}\cdot \mathrm{Ttod})$ represents the annualized cost of the utility imports month m with the ToD tariff, $({\Sigma }_{\mathrm{m}}{\Sigma }_{\mathrm{m}\in \mathrm{s}}{\Sigma }_{\mathrm{p}}{\mathrm{TP}}_{\mathrm{s},\mathrm{p}}\cdot \mathrm{max}({\Sigma }_{\mathrm{u}\in \mathrm{eo}}{\Sigma {\mathrm{U}}_{\mathrm{Loadt}}^{\mathrm{Imp}}}_{\mathrm{m},(\mathrm{d},\mathrm{h}) \in \mathrm{p}}$) shows the charges for maximum demand charges in particular month m, season s, and period p. the $(\Sigma \left(\mathrm{FCC}\cdot \mathrm{Pur}+\mathrm{ VCC}\cdot \mathrm{Cap}\right)\cdot \mathrm{An})$ represent the total cost of solar PV including fixed and variable costs computed annually. The last part of the objective function relates to reliability ($\mathrm{\Sigma EENS}{ .\mathrm{ C}}_{\mathrm{EENS}}$) shows the cost incurred with loss of demand during the year.

The constraints of this objective function are as follows:

- Eq. (2) forced a balanced constraint between load, generation and energy imported from the utility.
- Eq. (3) forced constraint of maximum DER generation during import and export mode of operation.
- Eq. (4) forced capacity constraint of solar generation capacity and consumer energy purchasing decision, where M = arbitrarily large number.
- Eq. (5) forced solar PV generation constraint which shows the solar generation for self-consumption and export is less than or equal to the theoretical solar PV generation.
- Eq. (6) forced solar PV area constraint which limits the installation of solar panel numbers in the given area.
- Eq. (7) forced constraint of demand supplying during the outage period

Assumption

- The movement of clouds and other external factors that effecting PV output are not considered
- A single ToD tariff applies to the entire load.
- The deterministic approach is followed while gathering the required data.
- With the cap of solar PV integration to 20%, the voltage and thermal limits are not taken into account.
- The grid-connected solar panels are installed at every customer's premises and their generation capacity is aggregated at the distribution transformer level.

The two major components of VPP are DER and DSM, both these components are modeled as follows.

### Solar PV

Solar PV is a weather-dependent DER that is quite popular in countries located near the equator due to high solar irradiance throughout the year. The output of a solar cell is well dependent on different variables which are briefly described below:

$${\mathrm{PV}}_{\mathrm{t}}$$ = A ∗ ɳ * N *S_t_∀ t ∈ T.

ɳ = Efficiency of a solar panel (14.9%)

S_t_ = Solar irradiation (800 W/ m^2^)

t = Time step

N = Number of solar panels connected

A = Area occupied by solar panels

${\mathrm{PV}}_{\mathrm{t}}$ = Estimated output of solar PV.

Lifetime of the PV = 25 years

PV Inverter size = 1.1*solar capacity

It is assumed that the PV panels are fully reliable and integrated with an inverter of the same rating. The maximum grid integrated capacity of PV is capped to 20% of transformer capacity due to safety constraints. The numbers of solar panels are assumed to be installed by every industry consumer connected to the feeder through a distribution transformer.

### Loads

The load is supplied up from SCADA controlled 66/11 kV step-down substation that is located within the boundary of the division. In this research, the 11 kV industrial feeder on which the DERs are installed is selected for load assessment. The feeder's load profile is aggregated into a single operating profile and analyzed for different cases. The load data of the feeder is taken from the utility database.

$${\mathrm{Load}}_{\mathrm{t}}$$ =$${\mathrm{FlexL}}_{\mathrm{t}}$$ + $${\mathrm{IntrrL}}_{\mathrm{t}}$$  + $${\mathrm{EmgL}}_{\mathrm{t}}$$∀t ∈ T (kW).

${Load}_{t}$ = Total demand on the feeder 

$${\mathrm{FlexL}}_{\mathrm{t}}$$ = Schedulable demand.

${\mathrm{IntrrL}}_{\mathrm{t}}$= Non Schedulable demand.

$${\mathrm{EmgL}}_{\mathrm{t}}$$ = Emergency demand.

**Figure 1:**
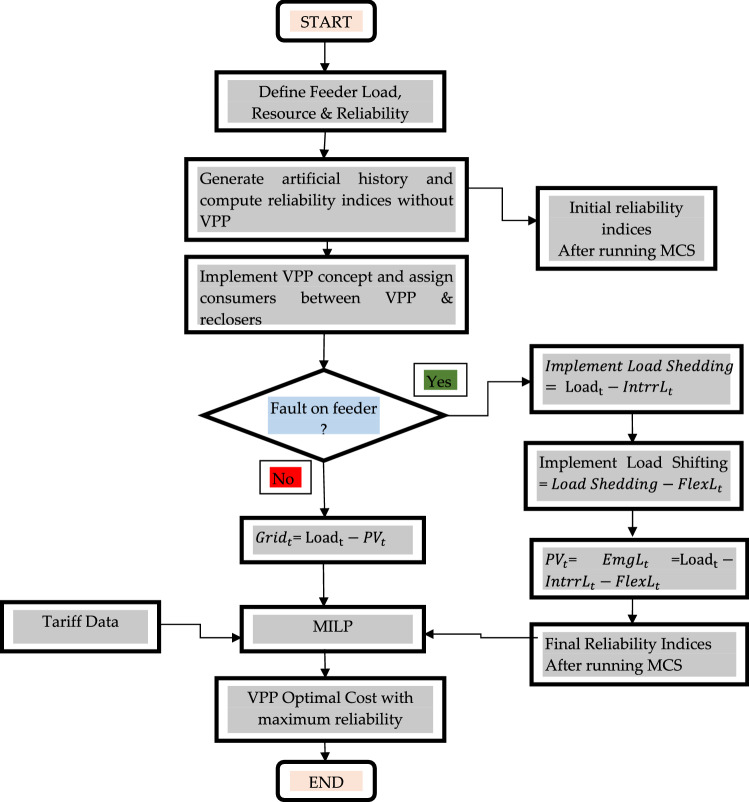
Workflow of MILP optimization.

The flexible load $${\mathrm{FlexL}}_{\mathrm{t}}$$ can be shifted from on-peak to off-peak period as tabulated in Table 4. The emergency demand $${\mathrm{EmgL}}_{\mathrm{t}}$$ is needed to supply at any cost even during a grid outage. On the other hand, the remaining demand is the inflexible demand, $${\mathrm{IntrrL}}_{\mathrm{t}}$$ which is neither flexible nor emergent. This can be curtailed during the outage period. The cost of load interruption is taken as $13.74/kWh which is the average loss value of the connected industries to this feeder. The workflow for the main body of the algorithm is illustrated in Fig. 1 below:-

The step-wise execution of MILP based VPP model is described as follows:

- Step1: Initialize the definition of various system components. The reliability and loading parameters of the feeder are also stated in this section.
- Step 2: The artificial failure history of network components is generated by MCS and reliability indices are computed without the VPP concept.
- Step 3: TheVPP concept is implemented followed by assigning the position of reclosers and the number of consumers at a particular location of feeder.
- Step 4: The feeder is evaluated in the given period for fault occurrence. If a fault is detected, intentional islanding is performed on the feeder, and electricity is dispatched through the DSM techniques such as load shedding and shifting. The emergency demand is dispatched through the PV. The updated reliability indices are computed with the VPP.
- Step 5: The initial healthy portion of the feeder is dispatched through the main grid.
- Step 6: The tariff data and updated reliability indices are fed to MILP optimization for optimal scheduling.
- Step 7: The optimal VPP cost is calculated which provides maximum reliability and techno-economic analysis has been done.

The study is further classified into three major cases for evaluating and comparing the reliability assessment with the VPP.

**Case 1: Base Case**

For setup reference reliability of the system, the base case is needed to be run. In this case, the VPP investment and reclosers are disabled.

**Case 2: With Reclosers**

The two automatic reclosers at different positions on the feeders are installed and the reliability of the system is determined. These reclosers isolate the faulty sections and energize the remaining portion of the feeder if it is located before the faulty section.

Case 3: With Reclosers and VPP

To further improve the reliability of the feeder, the DER is installed with the conjunction of automatic reclosers and the overall system reliability is accessed. The faulty portion is isolated and a healthy portion is energized either through the main grid or DER depending upon the location of the fault.

## Utility case study

The PSPCL is a power utility of the Punjab region (India) that has a monopoly on the generation and distribution of electrical energy in the state. The DER such as solar PV is the arising resource of power in the state aside from the conventional sources of energy. The state government also assists consumers of various categories in installing rooftop PVs at their premises through subsidies and monetary benefits^39^. To save energy bills for consumers and to have a sustainable energy future, the growth of this DER is escalating and contributes a significant share in the energy market in the coming years. The VPP concept could be an effective way of integrating the high capacity of DER in the utility grid. The potential of VPP in reliability improvement and minimizing cost for PSPCL is analyzed in this case study.

### Geographical and resource specification

**Figure 2:**
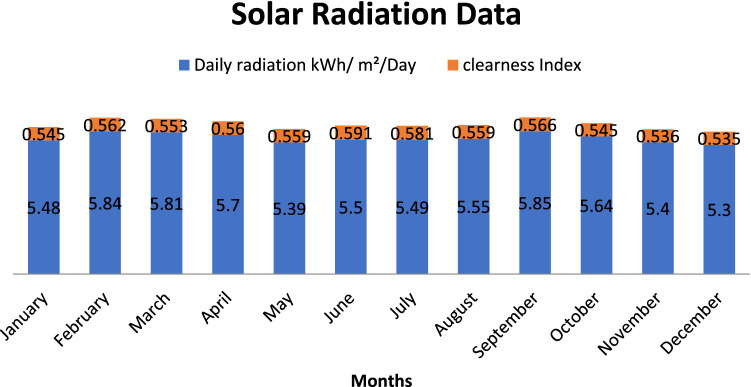
Annual average solar radiation and clearness index data.

The selected site is located under East division PSPCL (31.3260° N, 75.5762°) having abundant solar radiations throughout the year which makes it profitable to install PV panels in this area. The radiation data is downloaded from the National Renewable Energy Lab Database and illustrated in the Fig. 2 below.

### Load and reliability specifications

**Figure 3:**
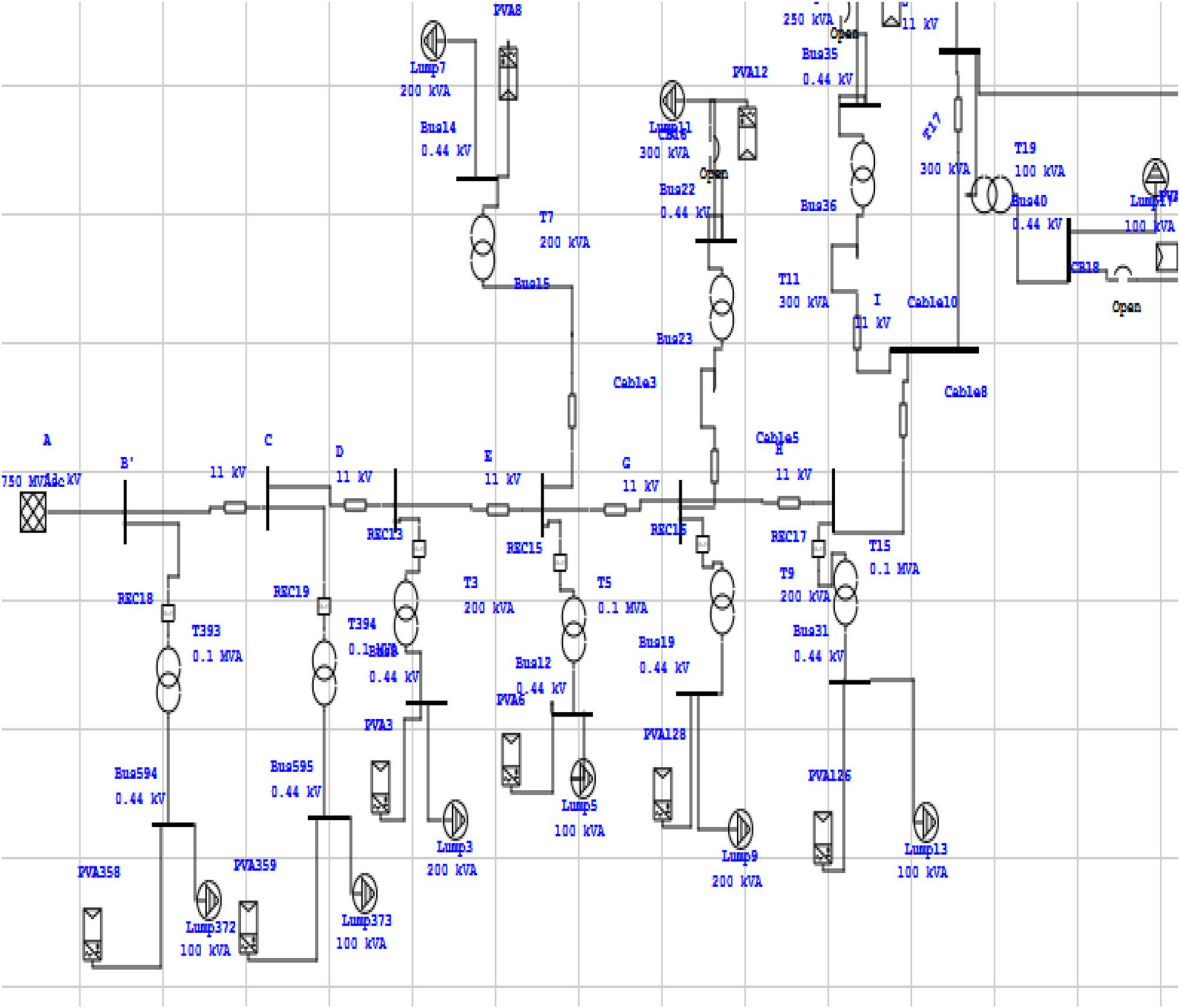
11 kV Industrial feeder 90 bus system.

For real-time VPP load evaluation, the 11 kV 90 bus Industrial feeder of the PSPCL is selected on which, the major load of furnace and punching machines is connected. A part of the one-line diagram of the 90 bus 11 kV industrial feeder designed in ETAP is shown below in Fig. 3. In addition, a complete 90 bus feeder is shown in the appendix as Fig. A1. The ETAP is a powerful tool for the reliability assessment used by both private and government organizations for system planning and improvement. In this paper, the ETAP is utilized to compute both energy cost and customer-oriented indices. The failure rate and MTTR are calculated by the substation data and by studying the fault history. The values significantly vary from the standard RBTS bus system. It includes the major components such as transmission lines, distribution transformers, solar PV, and step-down substation. The load and generating profiles are aggregated at a low voltage level and connected to an 11 kV feeder through a distribution transformer. It is assumed that all the distribution transformers installed on the feeder having grid-connected solar PV and their capacities are aggregated, but not more than 20% of the transformer capacity. The failure rate and MTTR of all the components of the feeder are shown in the appendix.

**Figure 4:**
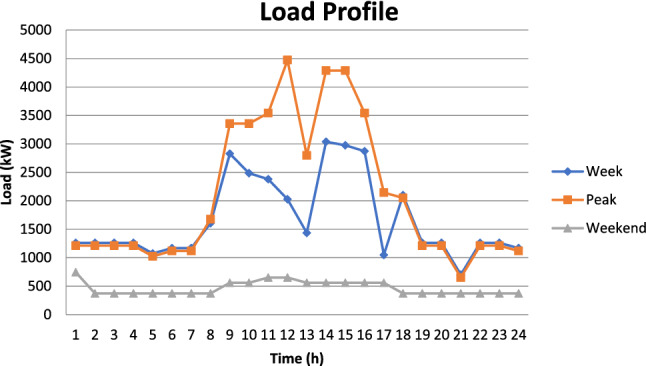
Load profile of industrial feeder.

To determine the influence of VPP, the numerous grid integrated DER located at different points of the feeder are interconnected into the feeding distribution transformer of their locality. The demand and generation profiles are aggregated at the feeding transformer level and the bi-directional flow of power is allowed to take place between the grid and consumers. The designed model computes the various variations in the parameters during different VPP scenarios for the complete year. For reliability assessment, the month of November is selected as there is a high demand in this particular month unlike in the residential feeder in which the load is the only peak during the summer season. The typical load Profile including week, weekend and peak day for November month of the industrial feeder is illustrated in Fig. 4.

The loading and reliability parameters of the Industrial feeder are shown below in Table 1.

**Table 1:** Industrial feeder technical parameters.

Feeder loading parameters		Feeder reliability parameters	
Annual average demand	1033 kW	**No. of distribution transformers on feeder**	47
Annual Peak demand	4288 kW	**Transformer Failure rate/year (**${\varvec{\uplambda}})$	0.15
Total annual energy consumption	97,18,850 kWh	**Transformer Mean time to repair (MTTR)**	0.5 h
Peak Month	November	**No. of feeder sections**	41
Total consumers on feeder	257	**Failure rate/yr (**${\varvec{\uplambda}})$** of feeder section**	0.5
Total length of feeder	2.174 KM	**Feeder section Mean time to repair (MTTR)**	1.5 h
Current capacity of feeder conductor	254 Amp	**No. of feeding substations**	1
Connected Load of feeder	9228 kVA	**Substation** **Failure rate/yr (**${\varvec{\uplambda}})$	0.6
Maximum demand	290 Amp/5518 kVA	**Substation** **Meantime to repair (MTTR)**	4 h

There is a significant difference between the annual average and peak demand which results in a lower load factor so there is a need for peak shaving which is possible through the VPP concept. The peak month for this industrial feeder comes to be November and energy consumption is also maximum in this case. The feeder's total length is divided into the number of sections to isolate the faulty section and allow the supply restoring of the remaining healthy feeder. The maximum demand on the feeder is already surpassing the current carrying capacity (254 Amp ACSR 48 mm^2^) of the feeder conductor which results in conductor breakdowns. However, the connected load is still higher than the maximum load and there is a possibility of an increase of peak load in the upcoming time. The failure rates of different components and their MTTR of the distribution line have been taken from the substation database. The numbers of transformers having different capacities are fairly distributed over the length of the feeder. The feeder section is part of the feeder between the mainline and transformer which has a fixed connected load and PV installation capacity. The feeding substation from which the distribution feeders have originated is also vulnerable to faults and having maintenance schedules for its operation. Therefore, it is also considered while doing reliability calculations.

### DER data

The VPP investment includes the capital cost of solar panels, inverter, and a static switch. In this research, the market available PV panel is considered without any government subsidy. The various technical and financial specifications of the VPP components are tabulated in Table 2 below^39^.

**Table 2:** Specification of VPP components.

Solar panel price ($/kW)	$ 1150
Solar panel lifetime	20 Years
Inverter price (100 kW)	$500
Solar panel peak eff	14.9%

The price of solar panels and inverters is highly varied from one region to another and expected to be a decline in the future with technological advancements. The polycrystalline technology-based solar panels are used in this study which is cheap but has lower efficiency than monocrystalline. The price of the inverter per kW is getting lower with a higher rating.

#### Tariff data

The ToD tariff^40^ was used for this study which is based on the power tariff 2019. The charges such as fixed and demand charges are also part of this tariff. The prime objective of this tariff is peak shaving to obtain a near-flat load profile which results in the deferral of utility network augmentation and declines consumer demand charges at the same time. The main features of this tariff are shown in Table 3 below:

**Table 3:** ToD tariff.

Date	Duration	Price/kWh
1–4–18 to 31–5–18	06:00 AM to 06:00 PM	$0.0907
	06:00PM to 10:00 PM	$0.0907
	10:00 PM to 06:00 AM	$0.0713
1–6–18 To 30–9–18	06:00 AM to 06:00 PM	$0.0907
	06:00PM to 10:00 PM	$0.1220
	10:00 PM to 06:00 AM	$0.0907
1–10–18 To 31–3–19	06:00 AM to 06:00 PM	$0.0907
	06:00PM To 10:00 PM	$0.0907
	10:00 PM To 06:00 AM	$0.0713

#### Flexible load data

The flexible or controllable load follows the generation which provides flexibility to the distribution grid with high penetration of DER. The PSPCL highly encouraged the consumers to shift their demand to off-peak periods by giving rebates through ToD tariff^41–43^. The maximum possibility of load shifting is found at weekends. The demand which could be controlled during various days is summarized in Table 4. The flexible load and shed load is calculated based on the historical energy data collected from the substation with and without the implementation of the Time of Day tariff. The percentage change in energy pattern becomes the base of our calculation of flexible load. The field surveys also have been done to know the load priority; moreover, the data which is downloaded from the smart meter shows the consumer demand pattern.

**Table 4:** Schedulable load data.

Feeder	Controllable load (percentage of aggregated load) during Peak day	Controllable load (percentage of aggregated load) during Weekday	Controllable load (percentage of aggregated load) during Weekend	Maximum load curtailment during peak hours	Maximum period
Industrial	18%	18%	32%	800 kW	12 h

## Results and discussions

After defining all the utility parameters, the developed model is simulated and the various indices are assessed in techno-economic terms.

### MCS results

After running the MCS with the 1000 sample years duration, the failures of different components of the feeder are simulated randomly. The prime step of MCS is to generate an artificial failure history of every component connected to the feeder. This failure history simulates the system for each hour of the year to find the possible failure and its consequence on all the load points of the feeder. The overall reliability results are summarized in Table 5 below.

**Table 5:** Reliability results of Industrial feeder.

Case	SAIFI	SAIDI	CAIDI	ASAI	EENS
Case1:base case with initial values	27.686	34.2946	1.2387	0.99609	37,045 kWh
Case2:with automatic reclosers	23.6684	31.3868	1.3261	0.99642	33,869 kWh
Case3:with automatic reclosers and VPP	9.5266	10.153	1.0657	0.99884	11,689 kWh

The above interruptions do not include the outages which are caused by scheduled maintenance or any erection work of the distribution network. The obtained results depict that the integration of reclosers and VPP into the network results in significant improvement in reliability indices such as SAIFI which is declined by 14.51% and 65.59% in case 2 and case 3 respectively. Without reclosers, there is no advantage of installing the DER as the faulty section cannot be isolated from the feeder and results in complete interruption of consumers. The flexibility of the system is also improved as an alternative supply is available from the DER.

### Techno-economic analyses

The reliability indices obtained are fed into MILP for evaluating the financial implications of VPP in reliability enhancement which includes calculating annualized and operational energy costs. The results from the proposed MILP optimization are shown in Table 6 below. After 1936 iterations, the best found the upper bound optimal value of the objective function is coming to be 0.097 and for lower bound it is 0.096.

**Table 6:** Proposed MILP optimization results.

Model solution status	8 integer solution
Solver termination condition	1 normal completion
Number of iterations	1936
Number of variables	726,673
Number of discrete Variables	50,526
Relative solver precision	0.02
Objval ub	0.097 best found
Objval lb	0.096
Equations	535,547
non zeros	1,458,906

In comparison to cases 1 and 2, the PV capacity 1916 kW is utilized in case 3 as a part of VPP integration which considerably declines the yearly energy cost by 55.26% of the feeder through dispatching the load by combining the effect of DER and DSM. The optimized operational cost is also reduced by 61% with DER penetration and DSM program such as load shifting which varies the consumer load pattern to make a balance between demand and supply. The cost of EENS with grid outage is also declined by the VPP implementation by 68%. The case in which the reclosers and VPP are included is found to be most economical in comparison to other configurations. The detailed techno-economic analyses of VPP are summarized in Table 7.

**Table 7:** Techno-economic analysis.

	Base case	With Reclosers	With Reclosers and VPP
PV capacity	–	–	1916 kW
Total annual energy costs	$18,91,000	$18,46,614	$8,46,016
Annual savings	0	$44,386	$10,44,984
Optimized operational cost	$18,91,000	$18,46,614	$7,37,578
Total electric costs	$18,90,513	$18,46,051	$7,25,280
Total annual electricity purchase	97,18,850 kWh	97,18,850 kWh	65,11,797 kWh
Total annual on-site generation	–	–	36,02,959 kWh
Load curtailment cost	$5,08,998	$46,53,60	$1,60,606

### VPP modes of operation

**Figure 5:**
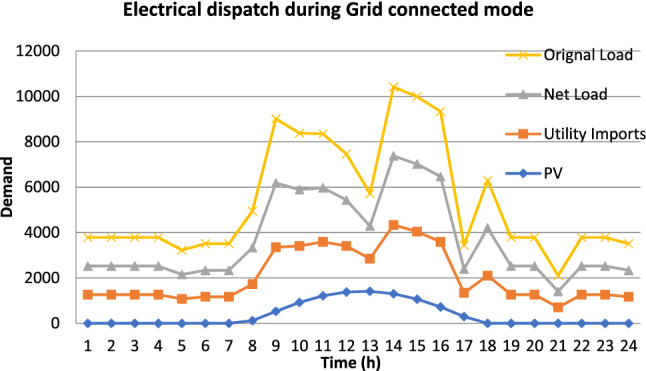
Electrical dispatch with VPP.

The VPP can operate in dual mode one is a grid-connected mode to minimize the energy cost. On the other side, it can be operated autonomously during the outage period and supply the maximum possible demand. The average daily electrical dispatch of the selected feeder by the grid-connected VPP concept for November month is illustrated in Fig. 5 below. The significant demand of the feeder is coinciding with the solar peak generation period hence it is possible to supply a major part of the load through VPP during that period.

**Figure 6:**
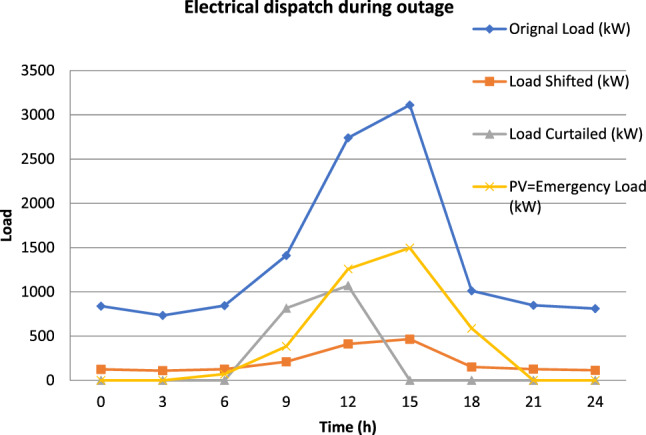
Electrical dispatch with VPP during an outage (Islanding mode).

The VPP plays an important role in the enhancement of reliability during an outage period in which the emergency load is supplied by a network of small-scale rooftop PVs and the remaining load is shifted or interrupted based on the supply priority. The load is also shifted to the off-peak period to lessen the load strain on the feeder during intentional islanding. For instance, the outage of an hour (09 to 10 h) is considered on the weekday of September month, Fig. 6 illustrates the electrical dispatch with VPP during an outage (Islanding mode).

During the outage of one hour, the demand of 211 kWh is shifted, 815 kWh curtailed and 384 kWh of emergency demand is met by PV. This operation of VPP considerably reduces the EENS and hence improved the overall reliability. To further analyze the demand implication of autonomous VPP, the section-wise load points of the 90 bus feeder are assessed and tabulated in Table 8 below. The branches of feeder are not considered in this evaluation and an outage of an hour is analyzed at various sections of feeders while assuming preceding sections of feeder section-wise autonomous response are isolated.

**Table 8:** VPP section-wise autonomous response.

Load section	Connected load (kW)	Running load (kW)	PV (kW)	Flexible load (kW)	Load shed (kW)
0–A	9228	4401	884	660	2857
A–B	9128	4354	874	653	2827
C–D	8928	4258	854	1339	2065
D–E	8528	4067	833	1279	1955
E–F	8328	3972	813	1249	1910
F–G	8128	3877	793	1219	1865
G–H	7898	3767	770	1184	1813
H–I	6878	3280	670	1031	1579
I–J	6678	3285	650	1001	1634
J–K	6578	3137	640	986	1511
K–L	6378	3042	620	956	1466
L–M	5378	2565	522	806	1237
M–N	5178	2469	502	776	1191
N–O	4678	2231	483	701	1047
O–P	4478	2136	462	671	1003
P–Q	4278	2040	441	641	958
Q–R	4178	1992	430	626	936
R–S	3778	1802	388	566	848
S–T	3678	1754	377	551	826
T–U	3478	1659	356	521	782
U–V	2978	1420	304	446	670
V–W	2778	1325	283	416	626
W–X	2578	1229	262	386	581
X–Y	2115	1008	214	317	477
Y–Z	1615	770	163	242	365
Z–A1	1552	749	156	232	361
A1–A2	1489	710	149	223	338
A2–A3	889	424	88	133	203
A3–A4	263	125	66	39	20
A4–A5	200	94	50	30	14
A5–A6	100	47	23	15	9

### Comparison between optimization techniques

The well-established optimization technique such as proprietary derivative-free algorithm (HOMER Optimization) is further used to validate and compare the proposed optimization results. HOMER Energy is a well-known simulation tool for designing microgrids and can be used for the economic analysis of VPP. In Homer, the various components are combined and subjected to constraints like a minimum renewable fraction, operating reserve, wind, and solar renewable output. In contrast to the proposed MILP model, the peak load profile is not utilized in the HOMER Pro software^44^. Both optimization techniques are implemented on the 90 bus industrial feeder VPP model to calculate the optimal operation cost. The results of the optimizations are described below in Table 9. The Homer Pro version 3.14 has been utilized to compare the results with DERCAM. The MILP optimization is more sophisticated and economical than HOMER Pro.

**Table 9:** Comparison of Different Optimization Techniques.

Optimization algorithm	Optimal operational cost	Execution time
Only grid	$18,91,000	00 S
HOMER Pro	$8,88,571	03.6 S
Proposed MILP	$7,37,578	15.1 S

## Conclusion

The prime objective of this research is to enhance distribution network reliability considering the financial implication of the grid outage and minimizing the energy cost. A case study of state power utility has been studied for the feasibility of VPP integration which is facing revenue loss due to frequent outages in its distribution network. The results acquired from the MCS revealed that there are significant improvements in the system reliability index such as EENS by 68% in the case of VPP deployment in comparison to the base case. These index values are further fed to the optimal model which is based on MILP is developed for evaluating the financial implications of reliability of the grid with the inclusion of reclosers, DER, and DSM. The techno-economic analysis of the optimization reveals that the operational cost is declined by 61% and also shows significant reductions in load curtailments. The results obtained through the optimization technique are compared with other popular algorithms such as the Proprietary Derivative-Free algorithm (Homer optimization) which also witnessed the effectiveness of the proposed optimization technique. It can be noticed that the VPP concept is found to be beneficial for both consumer and utility in the techno-economic prospectus. The proposed model has some limitations associated with the interruption duration and solar PV output. The Intrruption duration on the acccount of the scheduled maintenance and infrastructure development are not included while scheduling demand and computing outage duration. In addition to that, the solar PV generation profile used for computation is based on average monthly irradiation data available from NREL. These limititation could be addressed by including more comphrensive fault and outage history and using more accurate solar data from local renewable energy agencies. This study could be extended by the inclusion of storage devices and other types of DERs such as fuel cell, wind and biomass for dispatching the load during the fault period.

## Supplementary Information

Supplementary Information.

## Data Availability

All data generated or analyzed during this study are included in this published article and its supplementary information files.

## Abbreviations

ɳ: Efficiency of a solar panel (14.9%)
$${\mathrm{S}}_{\mathrm{t}}$$: Solar irradiation (800 $$\mathrm{W}/{\mathrm{m}}^{2}$$)
t: Time-step
N: Number of solar panels connected either in series or parallel
A: Area occupied by solar panels
$${\mathrm{PV}}_{\mathrm{t}}$$: Estimated output of solar PV
$${Load}_{t}$$: Total demand on the feeder
$${\mathrm{FlexL}}_{\mathrm{t}}$$: Schedulable demand
$${\mathrm{IntrrL}}_{\mathrm{t}}$$: Non schedulable demand
$${\mathrm{EmgL}}_{\mathrm{t}}$$: Emergency demand
s: Season (winter, summer including paddy season)
$${\mathrm{U}}_{\mathrm{Loadt}}^{\mathrm{Imp}}$$: Energy imported from the grid (kWh)
$${\mathrm{C}}_{\mathrm{EENS}}$$: Cost of not supplying demand ($/kWh)
$$\mathrm{EENS}$$: Expected energy not supplied (kWh)
$$\mathrm{Ttod}$$: Energy usage charges [$/kWh]
*Max*S: Maximum rating of solar PV [kW]
SPE: Theoretical peak solar conversion efficiency [%]
$${\mathrm{SRE}}_{\mathrm{m}.\mathrm{h}}$$: Solar radiation conversion efficiency of generation technology c, in month m, and hour h [%]
$$\mathrm{OMC}$$: Fixed annual O&M costs of solar PV [$/kW]
*MaxH*: Maximum annual operation hours for technology [hour]
NS: Number of units of solar PV installed
OS: Number of units of solar PV operating
$${\mathrm{PV}}_{\mathrm{t}}$$: Power generated by solar PV [kW
*Cap*: Rated output of solar PV [kW]
$${\mathrm{SI}}_{\mathrm{m},\mathrm{d},\mathrm{h}}$$: Solar insolation during specified time period of month, day and hour [kW / $${\mathrm{m}}^{2}$$]
$$\mathrm{VCC}$$: Variable capital cost of solar PV in [$/kW]
$$\mathrm{SA}$$: Available area for solar technologies [m^2^]
$$\mathrm{FCC}$$: Fixed capital cost of solar PV[$]
TTF: Time To Failure
Ui: Annual average unavailability
*Pur*: Consumer purchase binary decision of solar PV [0 or 1]
$${\mathrm{An}}_{\mathrm{i}}$$: Annuity factor for investments in technologies i
$${\mathrm{TF}}_{\mathrm{m}}$$: Tariff for fixed charges for using utility infrastructure in month m [$]
$${\mathrm{TP}}_{\mathrm{s},\mathrm{p}}$$: Maximum demand charges under the PSPCL tariff for season s and period p [$/kW]
P: Tariff period (on-peak, mid-peak, off-peak)
$$\uplambda$$: Failure rate/ Yr
PSPCL: Punjab state power corporation limited
NREL: National renewable energy laboratory
PV: Photovoltaic
HOMER: Hybrid optimization model for multiple energy resources
SCADA: Supervisory control and data acquisition
RMU: Ring main unit
ToD: Time of day
MILP: Mixed integer linear programming
ETAP: Electrical transient analyzer program
MCS: Monte carlo simulation
DG: Distributed generation
PSO: Particle swarm optimization
DER: Distributed energy resource
DSM: Demand side management
DER-CAM: Distributed energy resource customer adoption model
VPP: Virtual power plant
SAIFI: System average interruption frequency index
SAIDI: System average interruption duration index
CAIFI: Customer average interruption duration index
ASAI: Average service availability index
MTTR: Mean time to repair
MINLP: Mixed integer non-linear programming
TTR: Time to repair

## References

[1] Oest F, Radtke M, Blank-Babazadeh M, Holly S, Lehnhoff S (2021). Evaluation of communication infrastructures for distributed optimization of virtual power plant schedules. Energies.

[2] Do Prado JC, Qiao W, Qu L, Agüero JR (2019). The next-generation retail electricity market in the context of distributed energy resources: Vision and integrating framework. Energies.

[3] Battu NR, Abhyankar AR, Senroy N (2019). Reliability compliant distribution system planning using monte Carlo simulation. Electr. Power Components Syst..

[4] Trivedi R (2022). Community-based microgrids: Literature review and pathways to decarbonise the local electricity network. Energies.

[5] Sarmiento-Vintimilla JC, Torres E, Larruskain DM, Pérez-Molina MJ (2022). Applications, operational architectures and development of virtual power plants as a strategy to facilitate the integration of distributed energy resources. Energies.

[6] Royapoor M, Pazhoohesh M, Davison PJ, Patsios C, Walker S (2020). Building as a virtual power plant, magnitude and persistence of deferrable loads and human comfort implications. Energy Build..

[7] Sharma H, Mishra S (2021). Optimization of solar grid-based virtual power plant using distributed energy resources customer adoption model: A case study of Indian power sector. Arab. J. Sci. Eng..

[8] Sharma H, Mishra S, Dhillon J, Sharma NK, Bajaj M, Tariq R, Rehman AU, Shafiq M, Hamam H (2022). Feasibility of Solar Grid-Based Industrial Virtual Power Plant for Optimal Energy Scheduling: A Case of Indian Power Sector. Energies.

[9] Ndawula MB, Djokic SZ, Hernando-Gil I (2019). Reliability enhancement in power networks under uncertainty from distributed energy resources. Energies.

[10] Neto, A.C., Silva, M.G., Ieee, M. & Rodrigues, A.B. Impact of distributed generation on reliability evaluation of radial distribution systems under network constraints. 7–12 (2006).

[11] Ahmad S, Asar AU (2021). Reliability enhancement of electric distribution network using optimal placement of distributed generation. Sustain..

[12] Chiradeja P, Yoomak S, Ngaopitakkul A (2017). Optimal allocation of multi-DG on distribution system reliability and power losses using differential evolution algorithm. Energy Proc..

[13] Gong H, Ione DM (2021). Improving the power outage resilience of buildings with solar pv through the use of battery systems and ev energy storage. Energies.

[14] Battu NR, Abhyankar AR, Senroy N (2015). DG planning with amalgamation of economic and reliability considerations. Int. J. Electr. Power Energy Syst..

[15] Khalesi N, Rezaei N, Haghifam MR (2011). DG allocation with application of dynamic programming for loss reduction and reliability improvement. Int. J. Electr. Power Energy Syst..

[16] Hamzeh M, Vahidi B, Askarian-Abyaneh H (2015). Reliability evaluation of distribution transformers with high penetration of distributed generation. Int. J. Electr. Power Energy Syst..

[17] Tuffaha, T. & Almuhaini, M. Reliability assessment of a microgrid distribution system with pv and storage. *Proc. - 2015 Int. Symp. Smart Electr. Distrib. Syst. Technol. EDST 2015*, pp. 195–199, 2015, doi: 10.1109/SEDST.2015.7315206.

[18] Ahmad S (2017). Impact of distributed generation on the reliability of local distribution system. IOSR J. Electr. Electron. Eng..

[19] Chahkandi H, Tavakoli S, Ghadimi N, Korjani S (2019). Reliability based optimal allocation of distributed generations in transmission systems under demand response program. Electr. Power Syst. Res..

[20] Raghuwanshi SS, Arya R (2020). Reliability evaluation of stand-alone hybrid photovoltaic energy system for rural healthcare centre. Sustain. Energy Technol. Assessments.

[21] Pothireddy SS, Vuddanti KMR (2022). Impact of demand response on optimal sizing of distributed generation and customer tariff. Energies.

[22] Anvari-moghaddam A, Rahimi-kian A, Mirian MS, Guerrero JM (2017). A multi-agent based energy management solution for integrated buildings and microgrid system. Appl. Energy.

[23] Hemmati M, Mohammadi-ivatloo B, Abapour M (2020). Day-ahead profit-based reconfigurable microgrid scheduling considering uncertain renewable generation and load demand in the presence of energy storage. J. Energy Storage.

[24] Sierla S, Pourakbari-Kasmaei M, Vyatkin V (2022). A taxonomy of machine learning applications for virtual power plants and home/building energy management systems. Autom. Constr..

[25] Chen T (2021). Optimal demand response strategy of commercial building-based virtual power plant using reinforcement learning. IET Gener. Transm. Distrib..

[26] Asl SAF, Bagherzadeh L, Pirouzi S, Norouzi M, Lehtonen M (2021). A new two-layer model for energy management in the smart distribution network containing flexi-renewable virtual power plant. Electr. Power Syst. Res..

[27] Alabi TM, Lu L, Yang Z (2022). Data-driven optimal scheduling of multi-energy system virtual power plant (MEVPP) incorporating carbon capture system (CCS), electric vehicle flexibility, and clean energy marketer (CEM) strategy. Appl. Energy.

[28] Lin WT, Chen G, Li C (2021). Risk-averse energy trading among peer-to-peer based virtual power plants: A stochastic game approach. Int. J. Electr. Power Energy Syst..

[29] Li B, Yang F, Qi B, Bai X, Sun Y, Chen S (2022). Research on key technologies of P2P transaction in virtual power plant based on blockchain. IET Smart Grid.

[30] Abdullah WSW, Osman M, Kadir MZAA, Verayiah R, Aziz NFA, Rasheed MA (2021). Techno-economics analysis of battery energy storage system (bess) design for virtual power plant (VPP)–A case study in Malaysia. J. Energy Storage.

[31] Chaiyabut, N., & Damrongkulkamjorn, P. Impact of customer scattering on distribution system reliability with distributed generation. *IEEE Reg. 10 Annu. Int. Conf. Proceedings/TENCON*, pp. 568–573, 2010, doi: 10.1109/TENCON.2010.5686745.

[32] Xie S, Wang X, Qu C, Wang X, Guo J (2013). Impacts of different wind speed simulation methods on conditional reliability indices. Int. Trans. Electr. Energy Syst..

[33] Mohamed, A.T., Helal, A.A., & El Safty, S.M. Distribution System Reliability Evaluation in Presence of DG. *Proc. - 2019 IEEE Int. Conf. Environ. Electr. Eng. 2019 IEEE Ind. Commer. Power Syst. Eur. EEEIC/I CPS Eur. 2019*, 2019, doi: 10.1109/EEEIC.2019.8783657.

[34] Jikeng L, Xudong W (2010). Reliability evaluation for distribution system with distributed generation. Asia-Pac. Power Energy Eng. Conf. APPEEC.

[35] Billinton R, Wang P (1999). Teaching distribution system reliability evaluation using Monte Carlo simulation. IEEE Trans. Power Syst..

[36] Zeineldin, H., El-Saadany, E.F., & Salama, M.M.A. Intentional islanding of distributed generation. *2005 IEEE Power Eng. Soc. Gen. Meet.*, vol. 2, pp. 1496–1502, 2005, doi: 10.1109/pes.2005.1489218.

[37] Krishnan, G. & Gaonkar, D.N. Intentional islanding operations of distributed generation systems with a load shedding algorithm. *PEDES 2012 - IEEE Int. Conf. Power Electron. Drives Energy Syst.*, 2012, doi: 10.1109/PEDES.2012.6484267.

[38] Siddiqui AS, Marnay C, Edwards JL (2005). Effects of carbon tax on microgrid combined heat and power adoption. J. Energy Eng..

[39] Sharma H, Mishra S (2019). Techno-economic analysis of solar grid-based virtual power plant in Indian power sector: A case study. Int. Trans. Electr. Energy Syst..

[40] “PSPCL Tariff,” 2018. http://docs.pspcl.in/docs/sesalesto20180719161203100.pdf.

[41] Erdinc O, Paterakis NG, Catalaõ JPS (2015). Overview of insular power systems under increasing penetration of renewable energy sources: Opportunities and challenges. Renew. Sustain. Energy Rev..

[42] Kakran S, Chanana S (2018). Smart operations of smart grids integrated with distributed generation: A review. Renew. Sustain. Energy Rev..

[43] Eid C, Codani P, Perez Y, Reneses J, Hakvoort R (2016). Managing electric flexibility from distributed energy resources: A review of incentives for market design. Renew. Sustain. Energy Rev..

[44] Sharma, H. & Kaur, G. Optimization and simulation of smart grid distributed generation: A case study of university campus. doi: 10.1109/SEGE.2016.7589517 (2016).

