# Supplementary material for: Designing of multi-objective optimal virtual power plant model for reliability enhancement in radial network: a case study of Indian power sector
Source: Sci Rep. 2022 Aug 4;12:13382. doi: 10.1038/s41598-022-16389-8 (PMC9352725; doi:10.1038/s41598-022-16389-8)

Appendix:

Table.A1 ETAP Reliability database of the industrial feeder

**Lambda (Failure rate/ year)**	**MTTR (Hr)**	**Component**
7	4	1
0.0500000000000000	1.50000000000000	2
0.0500000000000000	1.50000000000000	3
0.0500000000000000	1.50000000000000	4
0.0500000000000000	1.50000000000000	5
0.0500000000000000	1.50000000000000	6
0.0500000000000000	1.50000000000000	7
0.0500000000000000	1.50000000000000	8
0.0500000000000000	1.50000000000000	9
0.0500000000000000	1.50000000000000	10
0.0500000000000000	1.50000000000000	11
0.0500000000000000	1.50000000000000	12
0.0500000000000000	1.50000000000000	13
0.0500000000000000	1.50000000000000	14
0.0500000000000000	1.50000000000000	15
0.0500000000000000	1.50000000000000	16
0.0500000000000000	1.50000000000000	17
0.0500000000000000	1.50000000000000	18
0.0500000000000000	1.50000000000000	19
0.0500000000000000	1.50000000000000	20
0.0500000000000000	1.50000000000000	21
0.0500000000000000	1.50000000000000	22
0.0500000000000000	1.50000000000000	23
0.0500000000000000	1.50000000000000	24
0.0500000000000000	1.50000000000000	25
0.0500000000000000	1.50000000000000	26
0.0500000000000000	1.50000000000000	27
0.0500000000000000	1.50000000000000	28
0.0500000000000000	1.50000000000000	29
0.0500000000000000	1.50000000000000	30
0.0500000000000000	1.50000000000000	31
0.0500000000000000	1.50000000000000	32
0.0500000000000000	1.50000000000000	33
0.0500000000000000	1.50000000000000	34
0.0500000000000000	1.50000000000000	35
0.0500000000000000	4	36
0.0500000000000000	4	37
0.0500000000000000	4	38
0.0500000000000000	4	39
0.0500000000000000	4	40
0.0200000000000000	4	41
0.0150000000000000	0.500000000000000	42
0.0150000000000000	0.500000000000000	43
0.0150000000000000	0.500000000000000	44
0.0150000000000000	0.500000000000000	45

0.0150000000000000	0.500000000000000	46
0.0150000000000000	0.500000000000000	47
0.0150000000000000	0.500000000000000	48
0.0150000000000000	0.500000000000000	49
0.0150000000000000	0.500000000000000	50
0.0150000000000000	0.500000000000000	51
0.0150000000000000	0.500000000000000	52
0.0150000000000000	0.500000000000000	53
0.0150000000000000	0.500000000000000	54
0.0150000000000000	0.500000000000000	55
0.0150000000000000	0.500000000000000	56
0.0150000000000000	0.500000000000000	57
0.0150000000000000	0.500000000000000	58
0.0150000000000000	0.500000000000000	59
0.0150000000000000	0.500000000000000	60
0.0150000000000000	0.500000000000000	61
0.0150000000000000	0.500000000000000	62
0.0150000000000000	0.500000000000000	63
0.0150000000000000	0.500000000000000	64
0.0150000000000000	0.500000000000000	65
0.0150000000000000	0.500000000000000	66
0.0150000000000000	0.500000000000000	67
0.0150000000000000	0.500000000000000	68
0.0150000000000000	0.500000000000000	69
0.0150000000000000	0.500000000000000	70
0.0150000000000000	0.500000000000000	71
0.0150000000000000	0.500000000000000	72
0.0150000000000000	0.500000000000000	73
0.0150000000000000	0.500000000000000	74
0.0150000000000000	0.500000000000000	75
0.0150000000000000	0.500000000000000	76
0.0150000000000000	0.500000000000000	77
0.0150000000000000	0.500000000000000	78
0.0150000000000000	0.500000000000000	79
0.0150000000000000	0.500000000000000	80
0.0150000000000000	0.500000000000000	81
0.0150000000000000	0.500000000000000	82
0.0150000000000000	0.500000000000000	83
0.0150000000000000	0.500000000000000	84
0.0150000000000000	0.500000000000000	85
0.0150000000000000	0.500000000000000	86
0.0150000000000000	0.500000000000000	87
0.0150000000000000	0.500000000000000	88
0.0150000000000000	0.500000000000000	89
0.0150000000000000	0.500000000000000	90

Figure.A1 Complete 90 Bus Industrial Feeder

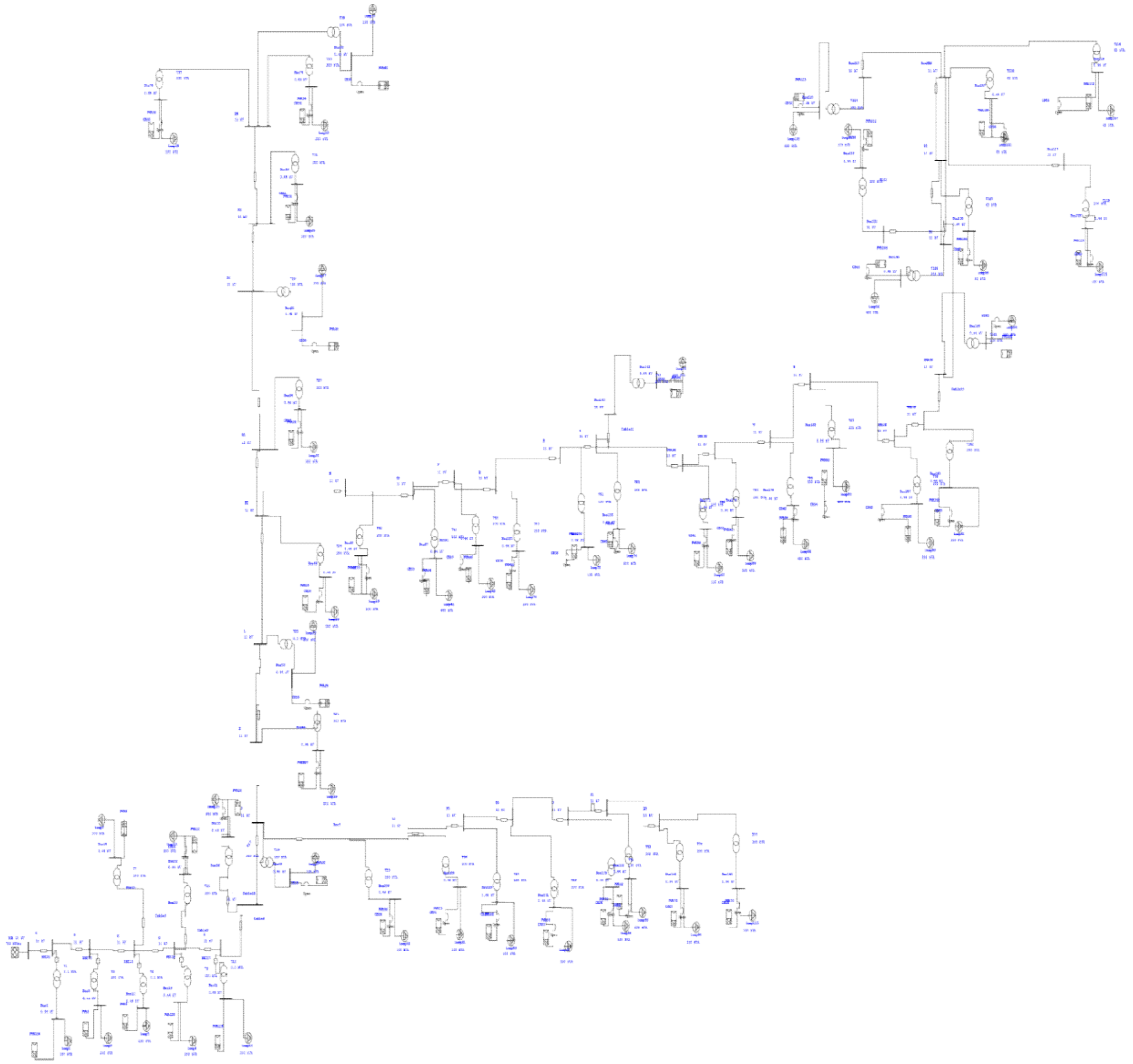

Supplement: Supplementary file 1 — Supplementary Information. [file 41598_2022_16389_MOESM1_ESM.pdf]
